# The impact of formal care provision on informal care receipt for people over 75 in England

**DOI:** 10.1371/journal.pone.0297157

**Published:** 2024-02-22

**Authors:** Eirini-Christina Saloniki, Olena Nizalova, Gintare Malisauskaite, Julien Forder

**Affiliations:** 1 Department of Applied Health Research, University College London, London, United Kingdom; 2 NIHR Applied Research Collaboration North Thames, London, United Kingdom; 3 Personal Social Services Research Unit, University of Kent, Canterbury, United Kingdom; University of South Florida, UNITED STATES

## Abstract

In this study, we examine the relationship between formal care provision and informal care receipt from within the household for people over 75 years old using data from the British Household Panel Survey between 1991 and 2009. To address potential concerns about endogeneity of formal care we use a ‘spatial lag’ instrumental variable. We find a negative and statistically significant effect of formal care provision on informal care receipt from within the household, suggesting a substantial degree of substitutability between these two modes of care. These findings provide grounds for estimates of savings in the cost of informal care enabled by spending on formal care, which is important in light of the effects of the caregiving burden and associated responsibilities on carer’s labour market participation.

## 1. Introduction

Informal (unpaid) care by family and friends is the mainstay of care provided for people with long-term care needs. In 2016, among those aged 75 and older in England, 55% received informal care and 7% used privately purchased care [1].

There has been a long-standing debate over the relationship between informal care and the provision of formal care. An increase in formal care services can lead to a decline in informal care provision, but there are implications for the (opportunity) costs to be (publicly) funded. Policy development in this area in England has tended to recognise the wider impact of care spending (in the situation of informal carers) in considering long-term care funding reform. Indeed, it is important to consider not only the effects of providing formal care on the direct recipient but also the effects for informal carers. Accordingly, this paper seeks to explore the impact of formal care on informal care need.

The direction of the relationship between formal and informal care is not straightforward and may depend on country-specific care eligibility criteria and institutional differences [2]. Most studies find a substitution effect between informal and formal care [3–6] while a few report a complementary relationship [7] (i.e. a ‘mixed responsibility’ effect whereby informal caregivers and formal care services take on specialised roles by providing the services that they deliver best).

Our study aims to explore how formal care provision affects the receipt of informal care from within the household. We utilise English survey data from the British Household Panel Survey (BHPS) between 1992 and 2009 [8] and employ instrumental variables estimations. Examples of informal care indicators identified in the literature include the average number of hours of informal care received from family members [9–11]. Alternatively, they are derived from questions in relation to looking after a person in need of help or assistance with activities of daily living [3,6,12,13] in a non-professional capacity, and we adopt this approach, characterised as the least problematic [14]. For formal care, we consider the use of services provided in the community relating for example to personal care, housekeeping and nursing.

The paper’s contribution to the existing literature is twofold: it explores the possibility of using (a) ‘spatial lag’ of formal care utilisation and (b) accounts for the discrete nature of both formal and informal care indicators in the estimations.

## 2. Methods

The data used in the study are available for secondary use from UK Data Service under an end user licence agreement, and do not require applying for institutional or any other ethical approval. Following previous studies [6], we use the question “Is there anyone living with you who is sick, disabled or elderly whom you look after or give special help to (for example, a sick or handicapped (or elderly) relative/husband/wife/friend, etc)?” and the BHPS personal number of the person they are caring for to identify those receiving informal care within a household. For formal care, we consider valid responses to whether the respondent used home help in the previous year.

We are interested in the propensity of an individual receiving informal care. We account for potential endogeneity in the relationship between home help and informal care due to unobserved omitted control variables, measurement error and reverse causality (e.g. individuals with high needs are likely to receive both modes of care), by estimating pooled instrumental variables (IV) models. We control for several socio-demographic and socio-economic characteristics such as age, gender, qualifications, marital status, self-assessed health, number of activities of daily living (ADLs), home ownership, household income (equivalised) and household size. Household size can limit the inside options of informal care receipt and in parallel increase the need for home help provision, hence we consider specifications with and without controlling for household size.

The IV models include a ‘spatial lag’ instrument for home help [15]: for each person in the dataset, we calculate the average home help utilisation of respondents in the dataset in the person’s region, excluding that person’s use of home help. We use this instrument as a signal of the relative generosity of each region, not directly linked to the respondents. The ‘spatial lag’ instrument may be valid for two reasons. The use of formal care by other people in the same region shall be correlated with a person’s use of these services due to common local authority policy factors in that market, yet there seems to be no reason to believe that it can affect informal care receipt other than through own formal care use. However, if there is a decline in the pool of informal carers due to population ageing [14], it may be that the receipt of formal care by others in the area relieves the burden from potential caregivers and thus directly affects the receipt of informal care. Therefore, as a sensitivity analysis, we further control for regional average employment to capture any local labour market characteristics. By comparison, one can argue that the ‘spatial lag’ shall be constructed at a lower geography level (e.g. local authority (LA)), which would however, come at the expense of less variation, threatening external validity and measurement error in the estimation of averages [16]. Indeed, when attempting the LA ‘spatial lag’ in BHPS, there were only a handful of individuals using home help (i.e., less than five) per LA in many LAs, which would make such analysis less accurate and meaningful. Thus, we did not proceed further with the LA ‘spatial lag’ in this analysis.

Existing literature (see [2] for a review) highlights the importance of social norms and attitudes towards unpaid care provision, with females more likely to provide certain types of care within the household. While males and females are more equally represented among older carers, early female exposure to informal caregiving may increase resilience and reduce the likelihood of home help use at an older age. This could signify a gender differential effect in the relationship between formal care provision and informal care receipt, which we explore in an additional analysis.

The older population are more likely to be frail and subsequently in need of formal and informal care [17]. Indeed, amongst those aged 65 and above only 6.8% receive home help and 8.4% receive within-household informal care, rising to 12.7% and 10.6% respectively for those aged 75 and above. Therefore, we restrict the sample to those aged 75 and above. The final sample size is 9,916 observations.

## 3. Results and discussion

Table 1 shows the average number of respondents receiving informal care within the household. One-tenth of respondents report receiving informal care from someone else in the household. On average, home help users are slightly less likely to receive informal care compared to those not using home help, but the difference is not statistically significant. When distinguishing by gender, male home help users are, on average, significantly more likely to receive informal care than those not using home help (0.17 versus 0.12); this is not the case for females (0.08 versus 0.10).

**Table 1:** Informal care receipt.

*People 75+*	Within household
Mean	0.11
Std. Dev.	0.31
*People 75+ using home help*	Within household
Mean	0.10
Std. Dev.	0.30
% of sample	12.70
*People 75+ not using home help*	Within household
Mean	0.11
Std. Dev.	0.31
% of sample	87.30
Observations	9,916

To account for the endogenous home help, we rely on the ‘spatial lag’ instrument, which we test for its relevance and validity. We first report findings from a pooled two-stage least squares (2sls) estimation, which allows for an extensive set of instrument-relevant tests. However, 2sls can be inconsistent as it allows probabilities to be estimated that are not between zero and one [18,19], which is crucial for the first-stage estimates. Further, it does not account for the discrete nature of informal and formal care variables, for which we estimate an extended IV model (i.e. probit estimation in both stages). The *eprobit* command in Stata 16 was used to run the extended IV model [20]. Despite the advantages of this model, it is significantly limited in diagnostics reporting–as a minimum, we test for the weakness of the instrument by manually calculating the F-statistic from the first stage.

In the first stage, we find that the average home help use by region (‘spatial lag’) is a strong predictor of home help (Table 2). The results from the under-identification test reject the null hypothesis indicating that the models are identified and the excluded instrument is relevant. The F-statistic from the first stage is over 10, indicating that the chosen instrument is strongly correlated with the endogenous home help.

**Table 2:** First-stage results.

	2sls	2sls	2sls	eprobit	eprobit	eprobit
	(1)	(2)	(3)	(4)	(5)	(6)
Home help ‘spatial lag’	0.788*** (0.203)	0678*** (0.189)	0.728*** (0.192)	4.041*** (1.065)	3.196*** (0.982)	3.833*** (1.105)
Female	-0.274 (0.226)	-0.237 (0.218)	-0.182 (0.222)	2.309** (1.048)	2.426** (1.019)	3.011*** (1.104)
Age	-0.041 (0.040)	-0.048 (0.039)	-0.045 (0.039)	0.436*** (0.152)	0.413*** (0.152)	0.440*** (0.163)
Age squared	0.000 (0.000)	0.000 (0.000)	0.000 (0.000)	-0.002** (0.010)	-0.002** (0.001)	-0.002** (0.001)
Married	-0.070*** (0.010)	-0.002 (0.012)	-0.071*** (0.010)	-0.526*** (0.071)	-0.029 (0.106)	-0.599*** (0.077)
Smoker	-0.017 (0.017)	-0.026* (0.015)	-0.026* (0.015)	-0.095 (0.105)	-0.146 (0.095)	-0.160 (0.101)
Age*Female	0.004 (0.003)	0.003 (0.003)	0.003 (0.003)	-0.025** (0.013)	-0.027** (0.012)	-0.034** (0.013)
Fair health	-	0.018** (0.017)	0.018** (0.009)	-	0.192*** (0.058)	0.199*** (0.059)
Poor/very poor health	-	0.030** (0.015)	0.082*** (0.017)	-	0.501*** (0.074)	0.503*** (0.075)
Sight problems (other than needing glasses to read normal size print)	0.052*** (0.015)	-0.001 (0.011)	0.030** (0.015)	0.253*** (0.066)	0.175*** (0.066)	0.157** (0.070)
Hearing problems	0.002 (0.011)	-0.001 (0.011)	-0.002 (0.011)	-0.008 (0.059)	-0.007 (0.059)	-0.028 (0.062)
Arms, legs, hands, feet, back or neck issues (including arthritis and rheumatism)	0.060*** (0.009)	0.013 (0.008)	0.015* (0.008)	0.400*** (0.054)	0.083 (0.054)	0.122** (0.056)
Skin conditions/allergies	0.038** (0.017)	0.026 (0.017)	0.027* (0.016)	0.195** (0.082)	0.148* (0.078)	0.152* (0.081)
Chest/breathing problems, asthma, bronchitis	0.046*** (0.013)	0.002 (0.012)	0.001 (0.013)	0.266*** (0.062)	0.057 (0.063)	0.038 (0.064)
Stomach/liver/kidneys or digestive problems	0.051** (0.016)	0.026* (0.015)	0.028* (0.015)	0.237*** (0.069)	0.099 (0.068)	0.123* (0.069)
Diabetes	-0.003 (0.016)	-0.016 (0.016)	-0.021 (0.016)	-0.012 (0.108)	-0.036 (0.098)	-0.129 (0.110)
Anxiety, depression or bad nerves, psychiatric problems	0.054*** (0.018)	0.022 (0.016)	0.025 (0.016)	0.293*** (0.073)	0.150** (0.072)	0.153** (0.070)
Migraine or frequent headaches	0.028 (0.023)	0.019 (0.023)	0.017 (0.023)	0.093 (0.101)	0.045 (0.107)	0.048 (0.110)
Heart/high blood pressure or blood circulation problems	0.031*** (0.009)	0.012 (0.009)	0.012 (0.009)	0.166*** (0.053)	0.058 (0.054)	0.069 (0.056)
Other health problems	0.043** (0.017)	0.006 (0.016)	0.007 (0.016)	0.278*** (0.078)	0.074 (0.074)	0.089 (0.075)
ADL count	-	0.034** (0.013)	0.033** (0.013)	-	0.299*** (0.057)	0.308*** (0.059)
ADL count squared	-	0.007* (0.004)	0.007* (0.004)	-	-0.012 (0.014)	-0.018 (0.015)
Degree and above^†^	0.017 (0.027)	0.000 (0.024)	0.018 (0.024)	0.073 (0.190)	-0.080 (0.202)	0.121 (0.193)
House owner	-	0.004 (0.012)	-0.007 (0.012)	-	0.028 (0.065)	-0.075 (0.068)
Household income (log)	-	0.043*** (0.012)	0.017 (0.012)	-	0.306*** (0.070)	0.110 (0.071)
Household size	-	-0.087*** (0.011)	-	-	-0.709*** (0.089)	-
North-south dummies	Yes	Yes	Yes	Yes	Yes	Yes
Wave dummies	Yes	Yes	Yes	Yes	Yes	Yes
Observations	9,916	9,916	9,916	9,916	9,916	9,916
F-statistic (weak instrument)	15.13	12.88	14.47	14.39	10.59	12.04
K-P rk LM statistic [chi-sq(1)] (under-id)	14.42***	12.38***	13.83***	-	-	-

^†^It includes University diploma/Foundation degree, University or CNAA first degree (e.g., BA, B.Ed, BSc) and University or CNAA higher degree (e.g., MSc, PhD).

*Note*: Clustered standard errors at the individual level are reported in parentheses.

***p<0.01

**p<0.05

*p<0.1.

In the second stage, we mostly find a statistically significant and negative effect of home help on informal care within household, suggesting that formal and informal care are substitutes (Table 3). The absence of a significant home help coefficient in the 2sls specification that controls for household size and the validity results from the first stage highlight the complex relationship between household size and both types of care, mediated by the interrelation between household size and income. For this reason, we use the specification without controlling for household size as the preferred one in what follows. The results (not reported here, available on request) are qualitatively similar, albeit larger in magnitude, when we further control for regional average employment to capture any local labour market characteristics.

**Table 3:** Effect of home help on within-household informal care receipt.

	2sls	2sls	2sls	eprobit	eprobit	eprobit
	(1)	(2)	(3)	(4)	(5)	(6)
Home help^†^	-0.340 (0.247)	-0.295 (0.227)	-0.461** (0.235)	-1.270*** (0.130)	-1.772*** (0.079)	-1.691*** (0.109)
Female	0.236 (0.231)	0.486*** (0.174)	0.324 (0.204)	0.523 (1.007)	1.273 (1.010)	0.937 (1.028)
Age	-0.020 (0.037)	-0.010 (0.030)	-0.025 (0.036)	0.049 (0.153)	-0.036 (0.165)	0.027 (0.171)
Age squared	0.000 (0.000)	0.000 (0.000)	0.000 (0.000)	0.000 (0.001)	0.001 (0.001)	0.000 (0.001)
Married	0.141*** (0.023)	-0.036* (0.019)	0.118*** (0.021)	0.712*** (0.091)	0.191** (0.080)	0.695*** (0.095)
Smoker	0.022 (0.017)	0.019 (0.014)	0.016 (0.016)	0.158* (0.094)	0.187** (0.091)	0.156* (0.094)
Age*Female	-0.003 (0.003)	-0.006*** (0.002)	-0.004 (0.003)	-0.005 (0.012)	-0.014 (0.013)	-0.010 (0.013)
Fair health	-	0.023*** (0.008)	0.027*** (0.009)	-	0.284*** (0.057)	0.229*** (0.052)
Poor/very poor health	-	0.100*** (0.023)	0.114*** (0.025)	-	0.628*** (0.074)	0.558*** (0.068)
Sight problems (other than needing glasses to read normal size print)	0.081*** (0.019)	0.044*** (0.014)	0.049*** (0.015)	0.376*** (0.064)	0.288*** (0.069)	0.252*** (0.067)
Hearing problems	0.031*** (0.011)	0.020** (0.009)	0.023** (0.010)	0.149*** (0.056)	0.100* (0.059)	0.128** (0.056)
Arms, legs, hands, feet, back or neck issues (including arthritis and rheumatism)	0.078*** (0.018)	0.015* (0.008)	0.012 (0.012)	0.463*** (0.058)	0.192*** (0.064)	0.127** (0.062)
Skin conditions/allergies	0.016 (0.019)	0.002 (0.015)	0.003 (0.017)	0.066 (0.080)	0.015 (0.082)	0.007 (0.082)
Chest/breathing problems, asthma, bronchitis	0.071*** (0.018)	0.008 (0.011)	0.012 (0.012)	0.340*** (0.058)	0.022 (0.063)	0.073 (0.061)
Stomach/liver/kidneys or digestive problems	0.045*** (0.020)	0.016 (0.014)	0.014 (0.016)	0.195** (0.067)	0.136** (0.074)	0.076 (0.074)
Diabetes	0.080*** (0.025)	0.050*** (0.018)	0.058*** (0.022)	0.352*** (0.099)	0.218** (0.093)	0.288*** (0.097)
Anxiety, depression or bad nerves, psychiatric problems	0.042** (0.021)	0.007 (0.014)	0.005 (0.015)	0.191** (0.078)	-0.014 (0.088)	-0.004 (0.074)
Migraine or frequent headaches	-0.005 (0.022)	-0.022 (0.018)	-0.015 (0.020)	-0.042 (0.107)	-0.081 (0.126)	-0.066 (0.117)
Heart/high blood pressure or blood circulation problems	0.023* (0.012)	-0.007 (0.008)	-0.006 (0.009)	0.121** (0.052)	-0.004 (0.055)	-0.028 (0.053)
Other health problems	0.099*** (0.022)	0.048*** (0.015)	0.047*** (0.016)	0.454*** (0.078)	0.231*** (0.084)	0.194** (0.078)
ADL count	-	0.067*** (0.014)	0.074*** (0.016)	-	0.621*** (0.064)	0.534*** (0.059)
ADL count squared	-	0.005 (0.004)	0.006 (0.009)	-	-0.048*** (0.016)	-0.040*** (0.015)
Degree and above^‡^	-0.049** (0.024)	-0.039 (0.024)	-0.078*** (0.028)	-0.355** (0.181)	-0.209 (0.211)	-0.534** (0.209)
House owner	-	0.014 (0.010)	0.040*** (0.011)	-	0.007 (0.071)	0.244*** (0.070)
Household income (log)	-	0.040*** (0.014)	0.105*** (0.013)	-	0.425*** (0.078)	0.746*** (0.072)
Household size	-	0.208*** (0.025)	-	-	1.026*** (0.078)	-
North-south dummies	Yes	Yes	Yes	Yes	Yes	Yes
Wave dummies	Yes	Yes	Yes	Yes	Yes	Yes
Observations	9,916	9,916	9,916	9,916	9,916	9,916

^†^The probability of receiving informal care conditional on using home help (eprobit models) is -0.429*** [SE 0.078] (Model 4), -0.447*** [SE 0.021] (Model 5) and -0.478*** [SE 0.035] (Model 6). ^‡^It includes University diploma/Foundation degree, University or CNAA first degree (e.g., BA, B.Ed, BSc) and University or CNAA higher degree (e.g., MSc, PhD).

*Note*: Clustered standard errors at the individual level are reported in parentheses.

***p<0.01

**p<0.05

*p<0.1.

In an additional analysis, we run the estimations separately by gender (Table 4). For males, the results from the first stage indicate that the model is under-identified and the excluded instrument is not relevant. On the opposite, for females, ‘spatial lag’ performs well in the first stage, pointing again to a substitution relationship between home help and informal care receipt in the second stage. In the absence of a significant effect for males and given the first-stage results, it is difficult to make any further comparisons by gender.

**Table 4:** Effect of home help on within-household informal care receipt by gender.

	Males		Females	
	2sls	eprobit	2sls	eprobit
*First stage*	(1)	(2)	(3)	(4)
Home help ‘spatial lag’	0.113 (0.261)	1.560 (1.825)	1.154*** (0.256)	5.194*** (1.352)
F-statistic (weak instrument)	0.19	0.73	18.90	14.75
K-P rk LM statistic [chi-sq(1)] (under-id)	0.19	-	17.53***	-
*Second stage*				
Home help^†^	-1.386 (3.752)	-1.702 (0.160)	-0.296* (0.180)	-1.595*** (0.178)
Observations	3,918	3,918	5,998	5,998

^†^The probability of receiving informal care conditional on using home help (eprobit models) is -0.332*** [SE 0.041] (Model 4).

*Note*: Each specification includes controls for age, age squared, marital status, smoker, general health, specific health problems, ADLs and ADLs squared, home ownership, household income (log), north-south dummies and waves dummies. Clustered standard errors at the individual level are reported in parentheses. ***p<0.01

**p<0.05

*p<0.1.

The substitution effects can be expressed in cost terms. Assuming 10 hours of informal care per week [21] at the minimum wage, the average weekly cost of informal care is £82.1. Applying a unit cost to the average hours of home help per week [22]–an upper bound of home care costs reported in [23,24]–we can estimate the impact of this spend in terms of (reduced) informal care costs. For example, giving home help to someone who did not receive it before incurs an average cost of £196, but reduces the cost of within-household informal care by £39 –as based on our estimated substitution effect of 0.478 from model (6), i.e. 0.478×£82.1. This is equivalent to a reduction in the cost of informal care of £0.20 for a £1 spent on long-term care per week on average.

While this work focuses on within-household informal care receipt, the BHPS also includes a question about help received with, for example, providing or cooking meals, helping with basic personal needs like dressing, eating or bathing, and financial help. A positive response to any of these questions can be used to measure informal care received by children living outside the household. This indicator is qualitatively different from our preferred measure in terms of whom one receives care from and the nature of tasks considered [25]. Further, it is available only in two waves (11 and 16), substantially restricting the overall sample size and impacting model identification and instrument validity. Indeed, results from a linear estimation (without IV) show no significant effect on home help while in the IV models ‘spatial lag’ substantially underperforms in the first stage (available on request). A combined indicator of any care would seem an obvious next step. However, it would require assuming that those who did not receive within-household informal care in waves other than 11 and 16 did not also receive outside household informal care–an assumption rather strong and impossible to justify.

## 4. Conclusion

Our estimation results suggest that formal and informal care are substitutes, which is consistent with earlier studies in this area [3,6,26]. The separate focus on gender does not influence the direction of this effect for females, though the degree of substitutability is smaller. The absence of a significant effect of formal care on informal care receipt for males precludes us from making any direct inferences about gender differential effects.

Our findings highlight the interdependency between formal and informal care and provide support for the existence of systems that seek to increase coordination between these two modes of care. Better coordination should increase efficiency, the scale of which is indicated by the magnitude of the substitution effect. Importantly, our findings also support the development of an economic case for spending on formal care, which can subsequently impact people providing informal care, for example, by reducing the caregiving burden and enabling their participation in the labour market.
